# Computational drug screening for drug-resistant tuberculosis: advances, challenges, and future perspectives

**DOI:** 10.3389/fphar.2026.1808051

**Published:** 2026-10-26

**Authors:** ShanShan Hu, Xiaoyu Li, Zahra Batool, Xin Zheng, Jiajia Dong, Chi Zhang, Yin Yang, Bairong Shen

**Affiliations:** 1 Department of Respiratory and Critical Care Medicine and Institutes for Systems Genetics, Frontiers Science Center for Disease-Related Molecular Network, West China Hospital, Sichuan University, Chengdu, China; 2 Department of Respiratory and Critical Care Medicine, West China Hospital, Sichuan University, Chengdu, China; 3 Department of Clinical Research Management, West China Hospital, Sichuan University, Chengdu, China

**Keywords:** AI-driven modeling, drug resistance, drug screening, *Mycobacterium tuberculosis*, tuberculosis

## Abstract

Tuberculosis (TB), caused by *Mycobacterium tuberculosis*, remains a major global health challenge, further exacerbated by the rapid emergence of drug-resistant strains. Despite continuous efforts, the discovery of novel anti-TB agents has lagged behind the evolution of resistance, largely due to the limitations of conventional experimental pipelines and the growing biological complexity of the pathogen. In recent years, computational drug discovery approaches—including virtual screening, artificial intelligence (AI)-driven modeling, and hybrid computational frameworks—have substantially expanded the chemical space accessible for early-stage anti-TB research. However, their translational success remains limited. This discrepancy reflects a fundamental mismatch between target-centric computational paradigms and the multi-scale biological constraints of *Mtb*, including permeability barriers, efflux mechanisms, metabolic state heterogeneity, lesion-level pharmacokinetics, and regimen-dependent therapeutic effects. This review critically evaluates current computational strategies for anti-TB drug screening, with particular emphasis on their performance under TB-specific biological and resistance constraints. We further explore computational approaches designed to address drug-resistant *Mtb*, including resistance-aware compound design, host-directed therapy, and combination regimen modeling. Finally, we explore key limitations in current computational pipelines, including challenges in data quality, biological integration, and regimen-level modeling, and propose a shift toward biologically grounded, regimen-aware, and systems-level frameworks. Bridging the gap between *in silico* prediction and clinical efficacy will require integrating multi-scale biological realism into computational pipelines, enabling more reliable translation of computational advances into effective anti-TB therapeutics.

## 1 Introduction

Tuberculosis (TB), a chronic airborne infectious disease primarily caused by *Mycobacterium tuberculosis (Mtb)*, remains the foremost cause of mortality from a single pathogen globally (Dheda et al., 2024). This ancient disease has plagued humanity for millennia, inflicting cumulative fatalities that surpass those of other historic pandemics, such as smallpox, the Spanish flu, and even COVID-19 (Fernandes et al., 2022). In response, the World Health Organization (WHO) has set an ambitious goal of eradicating TB by 2035, pursuing a 95% reduction in both incidence and mortality compared to 2015 levels (World Health Organization, 2023).

Currently, standard treatment follows a WHO-recommended combination chemotherapy regimen comprising four first-line drugs: rifampicin (RIF), isoniazid (INH), pyrazinamide (PZA), and ethambutol (ETH). This regimen is consecutively administered in fixed doses for 2 months during the intensive phase, followed by a 4-month continuation phase during which RIF and INH are taken daily (Dartois and Dick, 2024). While effective against drug-sensitive TB, the prolonged and widespread use of these drugs has driven the emergence of drug-resistant *Mtb* strains, including multidrug-resistant TB (MDR-TB), extensively drug-resistant TB (XDR-TB), and totally drug-resistant TB (TDR-TB) (Naidu et al., 2023). MDR-TB is defined by resistance to two staple front-line drugs, RIF and INH, requiring the administration of second-line drugs, such as fluoroquinolones, amikacin, capreomycin, and kanamycin—agents often associated with considerable toxicity and extended treatment durations of up to 2 years. XDR-TB, as redefined by the WHO in 2021, refers to MDR-TB strains additionally resistant to a fluoroquinolone and at least one Group A drug, such as bedaquiline or linezolid (World Health Organization, 2021). TDR-TB manifests the most severe form of resistance, with strains unresponsive to all tested antibiotics plus investigational agents in the discovery pipeline (Niculescu et al., 2024; Allué-Guardia et al., 2021). The escalating prevalence of drug-resistant TB has critically attenuated the efficiency of existing treatment protocols, contributing to rising mortality rates. Unfortunately, the pace of discovering novel anti-TB drugs has not kept up with the rapid emergence of resistance (Aljeldah, 2022). This pernicious gap highlights an urgent need for innovative strategies to accelerate the development of effective anti-TB agents, thereby ensuring the continuous replenishment of the current drug pipeline with compounds capable of handling the evolving threat of drug resistance (Sadybekov and Katritch, 2023).

In this context, computer-aided drug design (CADD) approaches have emerged as powerful tools to accelerate early-stage screening, reduce experimental cost, and improve hit identification efficiency. Techniques such as virtual screening (VS), AI-driven modeling, and hybrid computational frameworks have been widely applied in various therapeutic areas. However, despite these methodological advances, the translational success of CADD in TB remains limited, particularly in the context of drug-resistant TB. Conventional anti-TB drug screening approaches cannot fully meet the requirements for developing therapeutics against resistant strains. Drug-resistant strains exhibit complex resistance mechanisms and distinct biological characteristics, which introduce additional challenges in the design of screening models, evaluation criteria, and candidate prioritization strategies. Therefore, computational drug screening approaches specifically tailored to drug-resistant strains are urgently needed to improve the identification of therapeutics capable of overcoming resistance.

A fundamental bottleneck in conventional CADD-based anti-TB screening lies in the prevailing assumption that high-affinity identification of target-binding molecules inherently ensures therapeutic efficacy. While this assumption may suffice in many disease contexts, it often collapses in the face of TB’s complex, multi-scale biological constraints. These include the uniquely impermeable mycobacterial cell envelope, active efflux systems, intracellular survival within hostile macrophage microenvironments, and the metabolic heterogeneity of non-replicating persistent bacilli (Alsayed and Gunosewoyo, 2023; Nangpal et al., 2025). Consequently, computational hits with potent biochemical inhibition frequently fail to achieve whole-cell bactericidal activity or survive the transition to *in vivo* models.

In this review, we critically evaluate the performance of CADD in the context of TB-specific biological and translational constraints. We systematically analyze virtual screening, AI-driven modeling, and hybrid computational approaches, highlighting where and why these methods succeed or fail in TB drug screening. Furthermore, we discuss an emerging paradigm shift from target-centric discovery toward regimen-centric design and underscore key challenges related to data quality, model generalizability, and cross-scale integration. Addressing these challenges will be essential for improving the translational utility of computational drug screening strategies for drug-resistant TB.

## 2 Biological and translational bottlenecks of TB drug efficacy in CADD

Unlike many other bacterial pathogens, *Mtb* imposes a multi-scale system of biological and translational barriers that fundamentally constrain the effectiveness of CADD. These constraints—spanning target vulnerability, cellular permeability, physiological state transitions, and lesion-level drug distribution—are the primary determinants of translational success (Sikandar and Xing, 2025; Farhat et al., 2024). Crucially, the limited clinical translation of TB computational models often stems not from inadequate modeling but from a systematic mismatch between target-centric optimization and the multi-layered biological reality of *Mtb* infection. As a result, compounds that perform well *in silico* frequently fail to translate into whole-cell activity or *in vivo* efficacy.

### 2.1 Target vulnerability and permeability constraints

A key limitation in TB drug discovery is that target essentiality does not necessarily imply pharmacological vulnerability. Moreover, the mycolic acid–rich cell envelope and active efflux systems severely restrict intracellular drug accumulation (Poulton and Rock, 2022). These factors decouple binding affinity from effective target engagement, yielding a high rate of false positives in CADD pipelines that rely solely on structural or biochemical optimization.

### 2.2 Physiological state and metabolic adaptation

*Mtb* dynamically adapts to host-induced stresses (e.g., hypoxia, acidic pH) by entering non-replicating or dormant states, accompanied by extensive metabolic rewiring (Modlin et al., 2021). This state transition alters target dependencies, rendering many growth-associated targets conditionally irrelevant. Consequently, biochemical potency does not reliably translate into bactericidal activity, limiting the predictive value of models that do not account for state-dependent physiology.

### 2.3 Spatial and lesion-level constraints

TB granulomas exhibit pronounced spatial heterogeneity, with necrotic caseum forming diffusion-limited regions. This results in lesion-specific pharmacokinetic–pharmacodynamic mismatches, where compounds with favorable systemic exposure often fail to reach therapeutic concentrations at the infection site (Pienaar et al., 2017; Strydom et al., 2019). Such spatial constraints are rarely captured in conventional CADD frameworks, contributing to the divergence between predicted and observed *in vivo* efficacy.

### 2.4 Additional translational constraints

Additional TB-specific factors further complicate the clinical translation of CADD predictions. These include the requirement for intra-bacterial activation of prodrugs (e.g., INH) and the clinical necessity for multi-drug regimens (Ou et al., 2026). Importantly, optimization at the single-compound level does not directly translate into regimen-level efficacy, meaning that CADD outputs must ultimately be evaluated within a system-level biological and pharmacological framework.

## 3 Advances in computational modeling approaches for anti-TB drug screening

Drug screening is a direct strategy for identifying novel compounds to replenish the anti-TB drug pipelines in the context of rising drug resistance. Advances in computational modeling have progressed from virtual screening to AI-driven modeling and further into hybrid frameworks that integrate diverse computational techniques. Importantly, however, most reported studies in this area remain confined to *in silico* prediction, lacking subsequent whole-cell or *in vivo* validation. This section systematically reviews recent advances in three major computational modeling approaches: virtual screening, AI-driven modeling, and hybrid computational approaches, with emphasis on their methodological capabilities and inherent limitations in the context of TB drug discovery. Representative studies are summarized and critically evaluated in Table 1, detailing target classes, data modalities, and the prevalent gaps in experimental validation.

**TABLE 1:** Computational modeling approaches for anti-tuberculosis drug screening.

Computational modeling approaches	Chemical compounds resources	Targets	Computational methods	Results	Year	References
Virtual screening	17 designed isoxazolidinone derivatives	—	3D-QSAR	Showed predictive capability for structurally unrelated isoxazolidinone derivatives with micromolar antitubercular activity	2013	Friggeri et al. (2013)
	Manually curated database comprising 86 inhibitors of DHQase II	DHQase II	Activity cliff analysis; 4D-QSAR	Identified key structural regions amenable to optimization for enhanced antitubercular activity	2021	Miranda et al. (2021)
	NPASS database	FabD	Molecular docking; MD simulations; free energy calculation	Novel two hits and one known hit showing stable interactions with the target	2024	Sundararajan et al. (2024)
	Asinex database	Pks13	Molecular docking; MD simulations; entropy energy analysis; ADMET and pharmacokinetics analysis	Identified three drug-like compounds with strong binding energies and stable docked complexes	2023	Altharawi et al. (2023)
	DUD-E, Chembridge, Chemdiv, and Specs databases	InhA	Ensemble docking-based virtual screening; MD simulations	Identified four active compounds with new skeletons	2023	Zhang et al. (2023)
	Phytochemical library of 239 compounds	PS	Molecular docking; ADMET profiling; MD simulations	Screened out three plant-derived and identified their stability	2024	Chouhan et al. (2024)
	352 phytochemicals from 6 plant species	DXR	Molecular docking; MD simulations; ADMET analysis	Three identified compounds showing good stability and safety in the protein’s active site	2024	Islam et al. (2024)
	Asinex database	MurG (*Rv2153c*)	Homology modeling, MD simulations; energy-optimized pharmacophore modeling, high-throughput virtual screening, molecular docking	Identified 10 potential inhibitors and three of them as lead compounds with structural stability	2018	Saxena et al. (2018)
	ZINC 15 database	MraY	Homology modeling; e-pharmacophore; MD stimulations; ADMET analysis	Identified 15 potential inhibitors; three showed stable interactions with key residues	2022	Pandey et al. (2022)
	NPASS and SANCDB databases	RpsA	Pharmacophore modeling; Molecular docking; ADMET analysis; MD simulations	The finally selected compounds exhibit stronger binding interactions, lower deviations or fluctuations, and greater structural compactness compared to the reference compound	2024	Alotaibi (2024)
	Purchased from the vendor Specs compound library (http://www.specs.net)	DNA gyrase	Similar searching; molecular docking; MD stimulations	Screened 6 compounds; carbazole derivatives can be considered a new scaffold for inhibitors	2024	Pakamwong et al. (2024)
AI-driven Modeling	MLSMR, TAACF, and the kinase library from LifeChemicals	—	Cheminformatics clustering; Bayesian machine learning model	These Bayesian models successfully identified 7 out of 9 *Mtb*-active compounds reported in published studies, and 8 out of 11 active compounds from pharmaceutical screening	2014	Ekins et al. (2014)
	Manually curated data	—	Bayesian, RP Forest, RP Single Tree, and SVM models	Bayesian model has a positive predicted value (hit rate) > 77%	2016	Ekins et al. (2016)
	Manually curated data	—	Linear regression, GCN, ConvLSTM, and XGboost	Machine learning models, combined with chemical space visualization, can guide the design and target prediction of new anti-TB compounds	2022	Lane et al. (2022)
	CHEMBL database	—	An ensemble model of SVMs, RFs, XGBoost, and DNN	The model performed predictions with AUC of 0.842 and 0.942 for the 10-fold cross-validated training set and external test set	2021	Ye et al. (2021)
	TIBLE and ChEMBL databases	—	Graph-based signatures; machine learning models including RF, Extra Trees, Gaussian Process, SVM, Gradient Boosting, and XGBoost	Extra Trees has shown the best-performing algorithm, achieving correlation coefficients of up to 0.89 on cross-validation and 0.88 on independent blind tests	2020	Pires and Ascher (2020)
	ChEBI, ChEMBL, and DrugBank databases	—	Semantic knowledge graph, gene expression profiling, Bayesian model	Identified niclosamide inhibit *Mtb*	2024	Yang et al. (2024)
Hybrid computational approaches	A series of 20 benzimidazole derivatives	PrpR	ADMET analysis; Molecular docking; MD simulations; Machine learning-based sensitivity prediction	The designed 3 compounds against *Mtb* better inhibitory activity than INH	2021	Rajasekhar et al. (2021)
	Enamine, DUD-E, and BindingDB databases	TMK	3D-QSAR; pharmacophore model; Molecular docking; ADMET analysis; MD simulation; machine learning-based binding energy estimation	The proposed molecules could serve as crucial TMK inhibitors for controlling *Mtb.*	2024	Chikhale et al. (2024)
	Pharmit platform	MtDHQ	Molecular docking; deep learning-based binding affinity prediction; MD simulations	new putative inhibitors of MtDHQ and reinforce the potential application of artificial intelligence methods for drug design	2023	Souza et al. (2023)
​	BindingDB and ZINC databases	TMK	SVM, kNN, RF and GNB models; molecular docking; MD simulations	The predictive model with 87.2% accuracy and compared to the standard reference compound, the top three hits revealed greater stability and compactness	2024	Shahab et al. (2024)
	ChEMBL and DrugBank databases	DNA gyrase	GNN-based molecular representation; XGBoost model; molecular docking; MD simulations	Identified aldoxorubicin and quarfloxin showed potent inhibition of *Mtb* strain *H37Rv* and validated the direct binding of the two compounds to *Mtb* DNA gyrase	2024	Zheng et al. (2024)
	ChEMBL database	Fatty Acyl-CoA synthase	*Reinvent4* with deep-learning framework; molecular docking; deep-learning-based tool to predict binding affinity; ADMET analysis; MD simulations	four new compounds were identified as potential lead candidates with promising inhibitory effects on *Mtb* lipid metabolism	2025	Pawar et al. (2025)
	Commercially purchased	ethA, DNA gyrase, and rpoB	GC-MS; molecular docking; MD simulations; free energy analysis; ADMET analysis	Identified Sclareol has obeyed all the rules, and it might be a potential chemical to treat TB	2024	Ponnusamy et al. (2024)
	ZINC, DrugBank databases	embB	PPIN; Molecular docking; MD simulations; high-throughput virtual screening	confirms that Azilsartan and Dihydroergotamine form stable complexes with embB and can be used as potential lead molecules	2025	Krishnan et al. (2025)

QSAR, quantitative structure-activity relationship; /: no available data; DHQase II, dehydroquinate dehydratase II; FabD, Malonyl Coenzyme-A, acyl carrier protein transacylase; MD, molecular dynamic; Pks13, polyketide synthase 13; ADMET, absorption, distribution, metabolism, excretion, and toxicity; InhA, enoyl-acyl carrier protein reductase; PS, pantothenate synthetase; DXR, 1-deoxy-d-xylulose-5-phosphate reductoisomerase; MurG, undecaprenyl-diphospho-N-acetylglucosamine transferase; MraY, phospho-N-acetyl-muramyl-pentapeptide translocase; *Mtb*, *Mycobacterium tuberculosis*; RpsA, ribosomal protein subunit-1; RP, recursive partitioning; SVM, support vector machine; GCN, graph convolutional network; ConvLSTM, convolutional long-short term memory neural network; TB, tuberculosis; DNN, deep neural network; RF, random forest; PrpR, propionate catabolism operon regulatory protein; INH, isoniazid; TMK, thymidylate kinase; MtDHQ, 3-dehydroquinate dehydratase; *k*NN, *k*-nearest neighbor; GNB, gaussian naive base; GNN, generated neural networks; ethA, ethionamide activator; rpoB, RNA, polymerase β subunit; GC-MS, Gas Chromatography-Mass spectrometry; embB, arabinofuranosyltransferase B; PPIN, Protein-protein interaction networks.

### 3.1 Virtual screening

Early efforts in computational anti-TB drug screening primarily relied on traditional virtual screening (VS), a pivotal computational strategy extensively employed to identify potential anti-TB agents. VS operates under two paradigms: screening of large-scale libraries and scaffold-driven design.

#### 3.1.1 Drug screening based on large-scale chemical libraries

VS can be broadly categorized into ligand-based (LBVS) and structure-based (SBVS) approaches.

LBVS operates under the assumption that structurally or physicochemically similar molecules tend to exhibit comparable biological activity. It encompasses a range of techniques like quantitative structure-activity relationship (QSAR), pharmacophore modeling, and similarity-based searching. These methods infer potential bioactivity by analyzing molecular properties and comparing candidate compounds to earlier reported actives (Motamen and Quinn, 2020). Traditional LBVS approaches often rely on two-dimensional (2D) molecular representations (e.g., atom types, topological descriptors), which restrict their ability to accurately capture conformational flexibility or explore broader chemical space. To address these shortcomings, more advanced approaches such as three-dimensional QSAR (3D-QSAR) and four-dimensional QSAR (4D-QSAR) have been developed. While 3D-QSAR incorporates spatial conformations of ligands into model construction, 4D-QSAR further accounts for dynamic conformational changes of molecules, thereby offering a more nuanced view of structure–activity relationships and enhancing the predictive accuracy (Friggeri et al., 2013; Miranda et al., 2021).

SBVS is one of the most extensively adopted computational techniques in anti-TB drug screening. It focuses on well-characterized protein targets involved in critical biological pathways (e.g., *Mtb* cell wall biosynthesis, DNA replication, and energy metabolism) (Kuang et al., 2022) (Figure 1). *Mtb* possesses a unique and complex cell wall structure composed of mycolic acids, arabinogalactan, and peptidoglycan, essential for maintaining cellular integrity, mediating host adaptation, and sustaining long-term virulence (Capela et al., 2023). Representative examples include the enoyl-acyl carrier protein reductase InhA, a key enzyme in mycolic acid biosynthesis and the target of isoniazid; DNA gyrase, which is indispensable for DNA replication; and ATP synthase, targeted by bedaquiline to disrupt bacterial energy metabolism (Hasenoehrl et al., 2020; Locher et al., 2015; Rao et al., 2008; Vilchèze and Jacobs, 2019; Xu et al., 2022). However, treating TB targets as a uniform set for docking-based screening overlooks a key reality: they differ markedly in clinical validation, structural tractability, whole-cell translatability, and resistance liability. A functional hierarchy is therefore required. Clinically validated targets (e.g., InhA, DNA gyrase, ATP synthase) represent the most reliable class; here, structure-based approaches primarily serve as optimization tools and must explicitly address resistance mutations (Mdluli et al., 2015). A second category comprises promising but difficult targets (e.g., DprE1, Pks13), which, despite structural accessibility, often fail to translate at the whole-cell level due to permeability barriers, efflux, or metabolic compensation (Kuang et al., 2022; Teng et al., 2024); these require integrative multi-scale modeling beyond standard docking. A third class includes targets with recurrent computational attention but limited translational success, often driven by structural availability; for these, conventional active-site docking is unlikely to be productive, and alternative strategies such as protein–protein interaction targeting or polypharmacology should be considered. As summarized in Table 2, structural information alone is insufficient to predict druggability in *Mtb*; target prioritization must be guided by translatability within the multi-scale biological constraints that govern actual drug efficacy.

**FIGURE 1:**
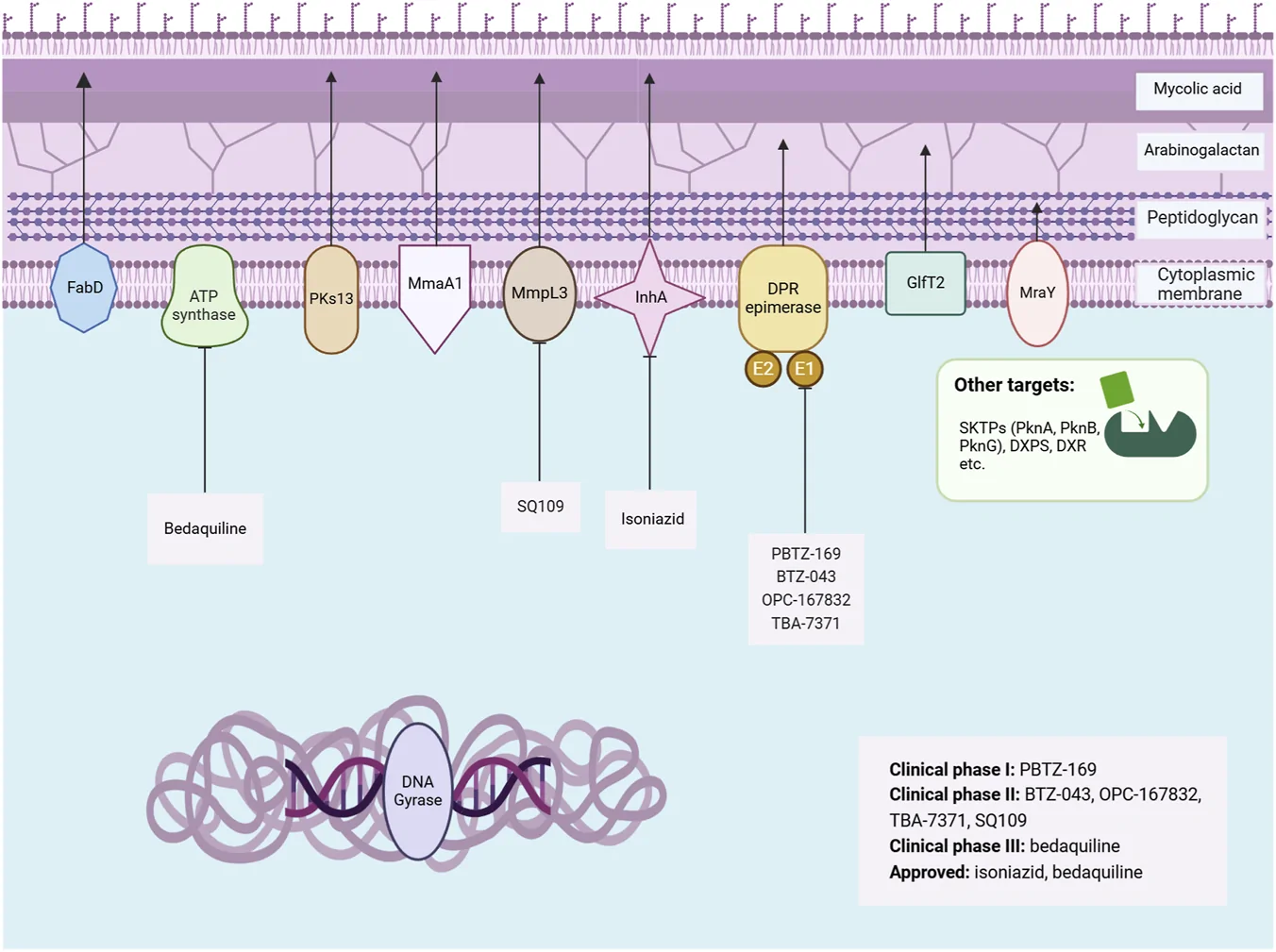
Primary well-established targets of screening anti-TB drugs. Figure created with www.BioRender.com. FabD, Malonyl Coenzyme-A Acyl carrier protein transacylase; PKs 13, polyketide synthase 13; MmaA1, mycolic acid methyltransferase 1; MmpL3, mycobacterial membrane protein large 3; InhA, enoyl-acyl carrier protein reductase; DprE1, flavoenzyme decaprenylphosphoryl-β-d-ribose 2′-oxidase; GlfT2, galactofuranosyltransferase 2; MraY, phospho-N-acetyl-muramyl-pentapeptide translocase.

**TABLE 2:** Stratification of TB drug targets for structure-based drug screening.

Target	Functional category	Structural confidence	Whole-cell translation	Resistance liability	Strategy suitability
Clinically validated targets (optimization-focused)					
InhA	Cell wall biosynthesis	High	Strong	High	Resistance-aware optimization
DNA gyrase	DNA replication	High	Strong	Moderate–high	Mutation-resilient optimization
ATP synthase	Energy metabolism	High	Strong	Moderate	Allosteric and resistance-aware optimization
embB	Arabinogalactan biosynthesis	Medium–high	Moderate	High	Combination strategies; resistance mitigation
ethA	Prodrug activation	Medium	Variable	High	Prodrug optimization; resistance circumvention
Promising but difficult targets (multi-scale modeling required)					
DprE1	Cell wall biosynthesis	High	Moderate	Moderate	Multi-scale and permeability-aware modeling
MmpL3	Lipid transport	Medium	Moderate	High	Combination strategies; resistance suppression
Pks13	Mycolic acid biosynthesis	Medium–high	Moderate	Unclear	Multi-scale modeling; target engagement validation
TMK	Nucleotide biosynthesis	High	Limited	Unclear	Multi-scale modeling; intracellular PK integration
MraY	Peptidoglycan biosynthesis	Medium	Limited	Moderate	Membrane-context structure-based design
FabD	Fatty acid biosynthesis	High	Moderate	Moderate	Pathway-level/multi-target strategies
PanK	CoA biosynthesis	High	Limited	Unclear	Conditional essentiality modeling
Low-translation/recurrent computational targets					
KasA	Fatty acid biosynthesis	High	Limited	Moderate	Reassessment; whole-cell validation
MurG	Peptidoglycan biosynthesis	High	Limited	Unclear	Permeability-aware reassessment
MtDHQ	Shikimate pathway	High	Limited	Low–unclear	Reassessment; translation-focused validation
EthR	Transcriptional regulator	High	Weak	Low	PPI modulation; adjuvant strategy
Lsr2	DNA-binding protein	Medium	Weak	Unclear	Non-classical inhibition; PPI targeting
RpsA	Translation (rescue pathway)	Medium	Uncertain	Unclear	Target validation; cautious prioritization

SBVS utilizes high-resolution three-dimensional (3D) structures of protein targets to model ligand–target interactions and estimate binding affinities (Motamen and Quinn, 2020). In the context of anti-TB drug screening, SBVS typically involves a multi-step pipeline integrating molecular docking and molecular dynamics (MD) simulations to systematically identify and optimize lead compounds (Figure 2A). This process generally consists of three stages: (1) Molecular docking to predict binding poses and estimate binding affinities; (2) MD simulations to examine the temporal behavior of discrete particles—such as molecules, ions, and clusters—by solving Newton’s equations of motion, thereby allowing dynamic assessment of interaction stability (e.g., hydrogen bonds, van der Waals forces) and binding energies; (3) ADMET evaluation to predict absorption, distribution, metabolism, excretion, and toxicity profiles of candidate compounds. However, such multi-layered computational workflows often rely on internally consistent metrics (e.g., docking scores, MD stability), which may reinforce each other without independent validation. As a result, they primarily support ligand-level prioritization rather than reliable prediction of biological activity. This integrative workflow enables efficient prioritization of compounds for experimental validation and has consistently yielded multiple candidates with favorable drug-like properties against *Mtb* infections, as documented in several studies (Altharawi et al., 2023; Sundararajan et al., 2024). Recent innovations have enhanced both the precision and flexibility of SBVS. For instance, ensemble docking-based virtual screening, which performs docking simulations across multiple protein conformations, accounts for protein flexibility and has successfully identified four compounds with novel scaffolds against *Mtb* (Zhang et al., 2023). Notably, natural products have garnered considerable attention in the search for novel anti-TB agents, given their structural diversity, accessibility, and comparatively lower synthesis costs. Two recent studies have successfully applied SBVS workflows to plant-driven compound libraries, identifying several natural product-based candidates with potential anti-TB activity (Chouhan et al., 2024; Islam et al., 2024). Even if such hits may not necessarily advance to final drug candidates, their unique chemical characteristics can offer a valuable source of novel scaffolds that can inspire drug discovery efforts (Newman and Cragg, 2012).

**FIGURE 2:**
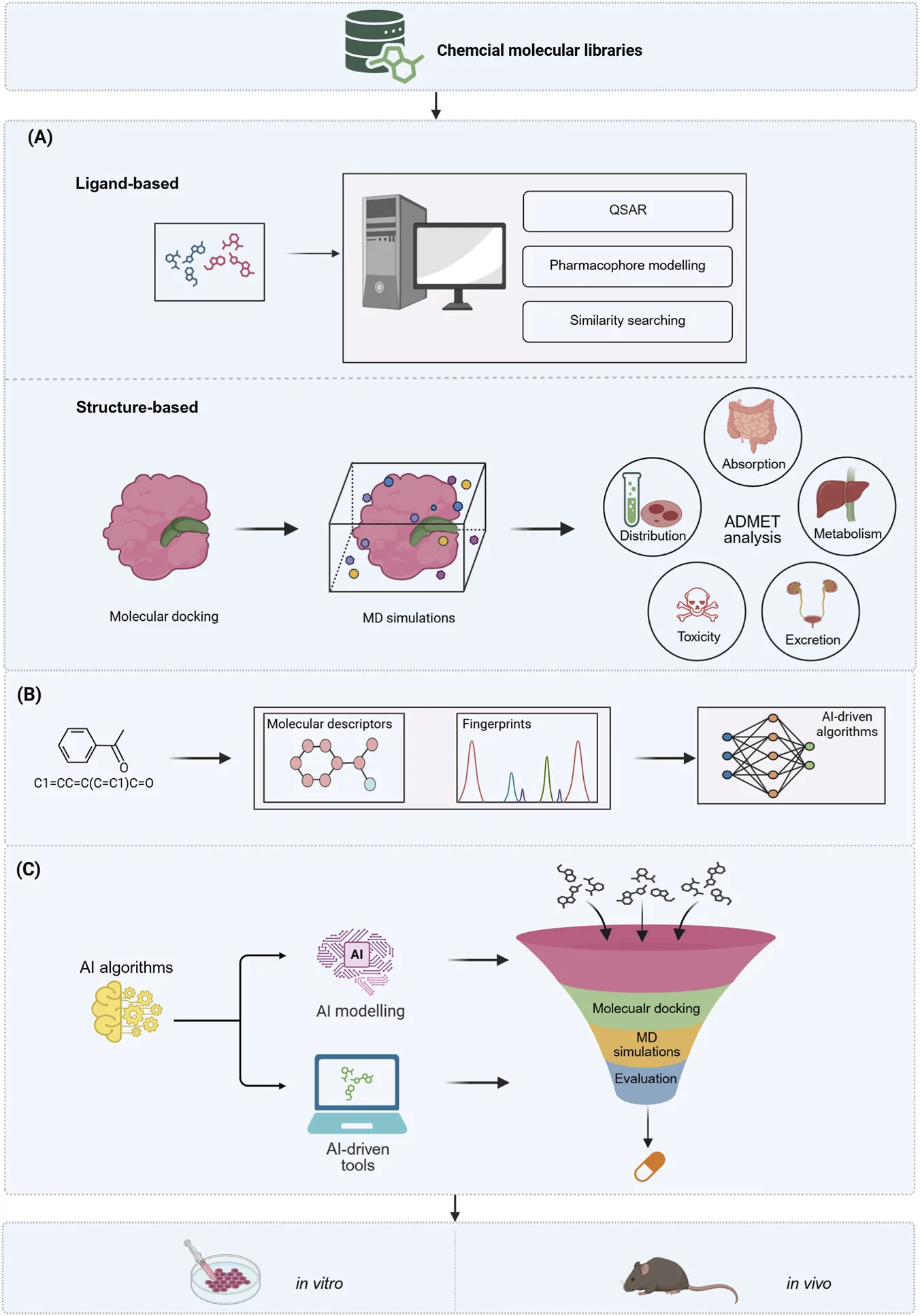
The overall workflow of computational approaches for anti-TB drug screening, integrating diverse *in silico* methods for candidate identification. The workflow includes three categories of computational strategies: **(A)** virtual screening approaches, including structure-based and ligand-based methods, used to prioritize compounds based on predicted target interactions; **(B)** AI-driven modeling approaches that apply machine learning or deep learning methods to enhance compound discovery and prioritization; and **(C)** hybrid computational approaches that integrate multiple *in silico* strategies to improve prediction robustness and screening efficiency. Figure created with www.BioRender.com.

Despite their utility, both LBVS and SBVS possess inherent limitations. LBVS is often constrained by the availability and diversity of known active ligands and lacks structural insight into target-ligand interactions. Conversely, SBVS requires high-resolution 3D target structures, which are not always available (Li Q. et al., 2023). In such cases, homology modeling can be adopted to generate putative 3D structures based on sequence similarity, although it carries the risk of structural inaccuracies when the template and target are evolutionarily distant (Pan et al., 2020). To overcome these limitations, hybrid approaches combining LBVS and SBVS have gained attention in anti-TB drug screening. These workflows often commence with LBVS to rapidly screen and prioritize candidate compounds, followed by SBVS to evaluate binding interactions and identify potential anti-TB candidates (Alotaibi, 2024; Pandey et al., 2022; Saxena et al., 2018).

In the screening pipeline, LBVS can be initially employed to narrow the chemical space by identifying a subset of promising candidates, which are subsequently subjected to SBVS for further validation (Pakamwong et al., 2024). This sequential strategy conserves computational resources for more intensive SBVS analyses and enhances the overall efficiency of the screening process.

Both LBVS and SBVS are fundamentally limited by their simplified representations of molecular recognition. More critically, they generally fail to capture the physiological realities of *Mtb*, such as the highly impermeable mycobacterial cell envelope and active efflux mechanisms. Consequently, high-scoring *in silico* hits frequently fail to translate into whole-cell minimum inhibitory concentrations.

#### 3.1.2 Drug screening based on chemical scaffolds

Beyond virtual screening of large-scale chemical libraries, an alternative strategy involves the intentional design and synthesis of compounds or their derivatives based on chemical scaffolds with documented pharmacological and biological activity, followed by molecular docking to evaluate their potential inhibitory effects. This scaffold-based design approach offers a targeted strategy for screening viable anti-TB leads. When selecting chemical scaffolds, it is imperative to prioritize structures associated with broad-spectrum biological activities. Prominent examples include azole-based scaffolds (such as oxadiazole and triazole) and the privileged chalcone scaffold (Figure 3A), both associated with broad-spectrum biological activities (e.g., anti-TB, antifungal, antiviral, and anticancer effects) (Alam, 2022; Biernacki et al., 2020; Sadineni et al., 2024). These scaffolds serve as pharmacophoric moieties for synthesizing novel hybrids via molecular hybridization techniques, with the goal of improving binding affinity and pharmacological efficacy against *Mtb* (Ibrahim et al., 2021; Sadineni et al., 2024; Sravanthi et al., 2024). Alternatively, scaffolds derived from approved antimycobacterial drugs provide well-defined chemical templates for the rational design of novel derivatives. For instance, the pyrazine ring (Figure 3B), a key pharmacophore of PZA, has inspired the development of various pyrazine-containing derivatives with novel anti-TB chemotypes (Gangarapu et al., 2020; Hassan et al., 2020). Compounds synthesized through such scaffold-based tactics typically incorporated at least one active pharmacophore unit, which may improve target binding affinity. Moreover, these compounds may exhibit dual-action mechanisms, potentially strengthening efficacy and alleviating adverse side effects (Liu et al., 2021).

**FIGURE 3:**
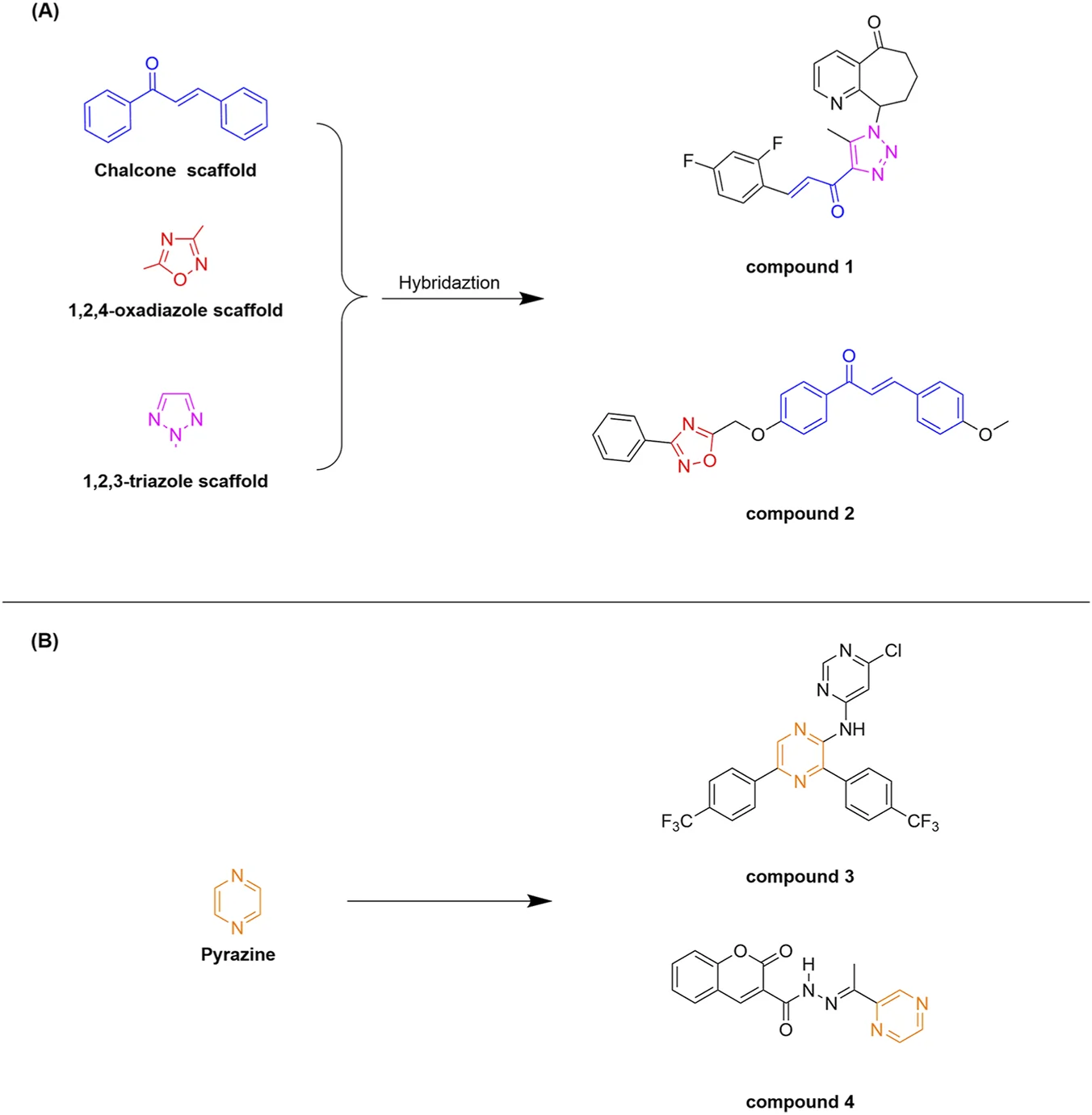
Examples of chemical structures of potential anti-TB agents with bioactive scaffolds. **(A)** Hybrids bearing chalcone and azole scaffolds (e.g., compound 1 and compound 2), **(B)** Designed compounds derived from the pyrazine ring (e.g., compound 3 and compound 4).

Overall, this scaffold-centric approach holds promise in mitigating the high attrition rates commonly encountered in later experimental validation stages, offering a more targeted and resource-efficient avenue for lead compound optimization. However, in practice, scaffold-based computational studies typically follow similar evaluation pipelines as VS (e.g., molecular docking and MD) and therefore share the same fundamental limitation: Predicted binding affinity does not equate to phenotypic efficacy against whole-cell or drug-resistant *Mtb* isolates.

### 3.2 AI-driven modeling

The exponential expansion of chemical libraries has, to some extent, increased the potential for early-stage drug discovery, yet it also introduced computational bottlenecks for VS workflows (Bender et al., 2021). Conversely, AI-driven modeling approaches, which learn predictive relationships directly from data, are especially well-suited for handling high-dimensional and heterogeneous datasets, including chemical structure and bioactivity data (e.g., DrugBank, ChEMBL and PubChem), drug–target interaction data (e.g., DrugBank and BindingDB), omics datasets, and literature-derived evidence. These diverse data sources enable AI models to capture complex biological and pharmacological relationships that are difficult to identify using conventional computational approaches. Consequently, AI-driven modeling offers a dynamic, data-centric alternative that can autonomously construct predictive frameworks, optimize parameters, and support decision-making (Schneider et al., 2020; Yang et al., 2019).

A key component of AI-driven modeling approaches, machine learning (ML) algorithms have been extensively applied in anti-TB drug screening. Ekins et al. (2014) employed cheminformatics-based clustering followed by Bayesian models to predict *Mtb*-active compounds, achieving an accuracy of 73%. Building on this work, the team later integrated *in vivo* data accumulated over seven decades across multiple laboratories, refining the model to improve the hit rate to 77% and enhancing its utility for clinical decision-making (Ekins et al., 2016). In 2022, they further developed a suite of ML-based models capable of predicting anti-TB activity, with expanded applications in novel drug design, target prediction, and data visualization analysis (Lane et al., 2022). These advances, spanning nearly a decade, underscore the growing role of AI in navigating previously uncharted chemical space to accelerate anti-TB drug screening. Importantly, AI-driven modeling bypasses explicit binding affinity evaluations, such as those performed by molecular docking, by directly predicting compound bioactivity from molecular features (Melo et al., 2021). For instance, a recent study extracted diverse molecular fingerprints to train an ensemble of classifiers, including support vector machines (SVMs), random forests (RFs), XGBoost, and deep neural networks (DNN), achieving high accuracy in identifying *Mtb*-active compounds (Ye et al., 2021).

Beyond activity prediction, AI-driven modeling has shown strong potential in generating molecular representations, a foundational aspect of computational screening (Figure 2B). Molecular representations convert structural and physicochemical features into numerical formats for predictive models. Traditionally, these representations have relied on fixed binary fingerprints, which denote the presence or absence of specific molecular substructures (Catacutan et al., 2024). However, it often fails to encapsulate the contextual details of molecular connectivity and lacks flexibility in training models for diverse predictive tasks. Since molecules can be inherently represented as graphs, with atoms and bonds corresponding to nodes and edges, recent studies have progressively attempted to leverage graph-based algorithms to depict 3D molecular topologies and model protein–ligand interactions. For example, Pires and Ascher (2020) proposed *mycoCSM*, a graph-based signature approach that proficiently captured 3D molecular configuration for predicting anti-TB activity. Likewise, the integration of semantic knowledge graphs with ML has enabled the identification of drug-disease associations, yielding 14 promising candidates from a dataset of 157 *Mtb*-related compounds (Yang et al., 2024).

Unlike SBVS, AI-driven modeling approaches do not explicitly model binding interactions but instead infer activity from learned patterns, enabling efficient screening across large chemical spaces while reducing dependence on structural data. Despite their predictive power, several limitations continue to restrict the broader application of AI in anti-TB drug screening. Dataset bias remains a major concern, as many models are trained on chemically narrow, imbalanced, or experimentally heterogeneous datasets that may inadequately represent the broader anti-TB chemical space. Consequently, strong performance on benchmark datasets does not necessarily translate into reliable prediction for structurally novel compounds or independent screening scenarios. Model interpretability poses an additional challenge, particularly for deep learning architectures whose predictions are often difficult to mechanistically rationalize in terms of molecular interactions or biological pathways. This limited transparency may hinder biological validation and reduce confidence in model-guided prioritization. Reproducibility also remains problematic due to inconsistent data preprocessing, variable evaluation protocols, and incomplete reporting of model architectures, training procedures, or code availability across studies.

### 3.3 Hybrid computational approaches

VS, despite its widespread application, does not capture the full complexity of molecular interactions, leading to reduced predictive accuracy. Conversely, AI-driven modeling excels at uncovering intricate patterns and nonlinear relationships within high-dimensional data, yet it heavily relies on high-quality annotated data and is prone to challenges such as overfitting and limited interpretability. These respective limitations underscore the growing value of hybrid computational approaches, which integrate the complementary strengths of VS, AI-driven modeling, and other computational tools to improve robustness and predictive power in anti-TB drug screening (Figure 2C). These frameworks integrate multiple computational tools to enhance candidate prioritization and streamline screening pipelines. Notably, AI has shifted from an auxiliary role to a central driver of the screening pipeline, refining chemical space exploration and dramatically improving screening efficiency.

Ultimately, while hybrid approaches improve computational robustness, they still inherit the fundamental limitation that all outputs remain *in silico* predictions without experimental grounding.

#### 3.3.1 Integration of virtual screening with AI-driven modeling

One prominent form of synergy lies in incorporating AI-driven tools as auxiliary tools within VS workflows. These off-the-shelf models based on ML or deep learning architectures can predict key pharmacological properties such as inhibitory activity, target binding affinity, and ADMET profiles, thereby expediting candidate prioritization and complementing molecular docking outcomes. For instance, the graph-based AI model *mycoCSM* was used to predict the inhibitory activity of designed benzimidazole derivatives, producing results consistent with SBVS and aiding in the identification of promising anti-TB candidates (Rajasekhar et al., 2021). Similarly, an AI model was applied to predict binding free energies of compounds targeting thymidylate kinase, providing valuable computational support for SBVS-based lead identification (Chikhale et al., 2024). In another case, the open-source AI-driven platform *DeepPurpose* was applied to estimate drug–target interactions. By integrating its output with docking results from two separate programs, researchers identified novel inhibitors with superior binding affinities compared to established compounds (Souza et al., 2023).

Beyond serving as auxiliary tools, AI models are increasingly embedded as core components of screening workflows. By narrowing the chemical search space and streamlining candidate selection, AI significantly enhances efficiency and reduces screening costs. For example, two recent studies trained ML models on large-scale compound databases to predict anti-TB activity, subsequently guided by molecular docking and MD simulations, thereby enhancing screening efficiency (Shahab et al., 2024; Zheng et al., 2024). Furthermore, *Reinvent4*, an AI-powered *de novo* drug design platform, was employed to generate novel inhibitors targeting fatty acyl-CoA synthetase, a key enzyme in *Mtb* fatty acid biosynthesis. Follow-up molecular docking and binding free energy analysis identified four lead candidates, demonstrating the potential of generative AI in combination with SBVS (Pawar et al., 2025).

#### 3.3.2 Integration of virtual screening with analytical tools

Hybrid computational approaches have also benefited from the incorporation of diverse analytical tools alongside VS to further enhance predictive accuracy and mechanistic understanding. For example, mass spectrometry, renowned for its ability to resolve chemical substructures with high precision, has been integrated into SBVS workflows to improve compound characterization and structural annotation (Ponnusamy et al., 2024). In another study, SBVS was integrated with protein–protein interaction (PPI) network analysis to infer functional relationships among potential targets, providing mechanistic insight and supporting rational target identification (Krishnan et al., 2025).

### 3.4 Comparative analysis and translational challenges

Collectively, LBVS, SBVS, AI-driven, and hybrid computational approaches provide complementary capabilities across anti-TB drug discovery pipelines (Table 3). LBVS offers computational efficiency and rapid exploration of compounds with known chemical similarity but is constrained by dependence on existing active ligands and limited novelty discovery. In contrast, SBVS leverages structural information to provide mechanistic insights and facilitate scaffold discovery, although its predictive performance depends heavily on target structural quality and scoring accuracy. AI-driven modeling approaches further expand screening capacity by learning complex structure–activity relationships and supporting large-scale prediction and molecular design, yet remain vulnerable to data bias, limited interpretability, and generalizability concerns. Hybrid computational frameworks increasingly integrate these complementary strategies to improve screening robustness and candidate prioritization.

**TABLE 3:** Comparative overview of ligand-based, structure-based, AI-driven, and hybrid virtual screening approaches.

Computational modeling methods	Core principle	Strengths	Limitations	Practical applications
Ligand-Based Virtual Screening (LBVS)	Similarity to known ligands	Fast, low computational cost, independent of target structure	Requires known active ligands; limited scaffold novelty	Lead optimization, similarity search
Structure-Based Virtual Screening (SBVS)	Target structural interaction	Enables novel scaffold discovery; mechanistic insight	Depends on structural accuracy; docking/scoring errors	Target-driven screening, hit identification, binding mode analysis, structure-guided optimization
AI-Driven Virtual Screening	Predictive modeling based on machine learning	Handles large datasets; captures nonlinear patterns	Data dependency; interpretability challenges	Activity prediction, ADMET prediction, *de novo* design, hit prioritization
Hybrid strategies	Combination of multiple strategies	Improved robustness and predictive performance	Higher complexity and integration cost	Multi-stage virtual screening, precision hit prioritization

However, despite methodological differences, these approaches share a common translational limitation. Their outputs primarily reflect computational predictions rather than validated biological efficacy. In TB drug screening, this limitation is particularly significant because molecular recognition alone does not account for critical physiological barriers such as mycobacterial cell-envelope permeability, efflux activity, and metabolic adaptation. Consequently, promising computational hits frequently fail to demonstrate whole-cell or *in vivo* activity. Therefore, computational modeling should be viewed primarily as a prioritization and hypothesis-generation framework that requires systematic experimental validation for successful translation.

## 4 Computational modeling strategies against the multilayered resistance landscape

The alarming surge of drug-resistant *Mtb* strains has emerged as a formidable obstacle to effective TB treatment. Conventional large-scale drug screening often lacks specificity toward the evolving resistance mechanisms. To circumvent this bottleneck, resistance-oriented countermeasures have been proposed, primarily focusing on designing resistance-evasive compounds, host-directed therapy (HDT), and rational drug combination regimens (Figure 4). Computational modeling approaches, particularly AI-driven modeling, have thus become an indispensable toolkit for accelerating and optimizing these anti-resistance drug discovery pipelines.

**FIGURE 4:**
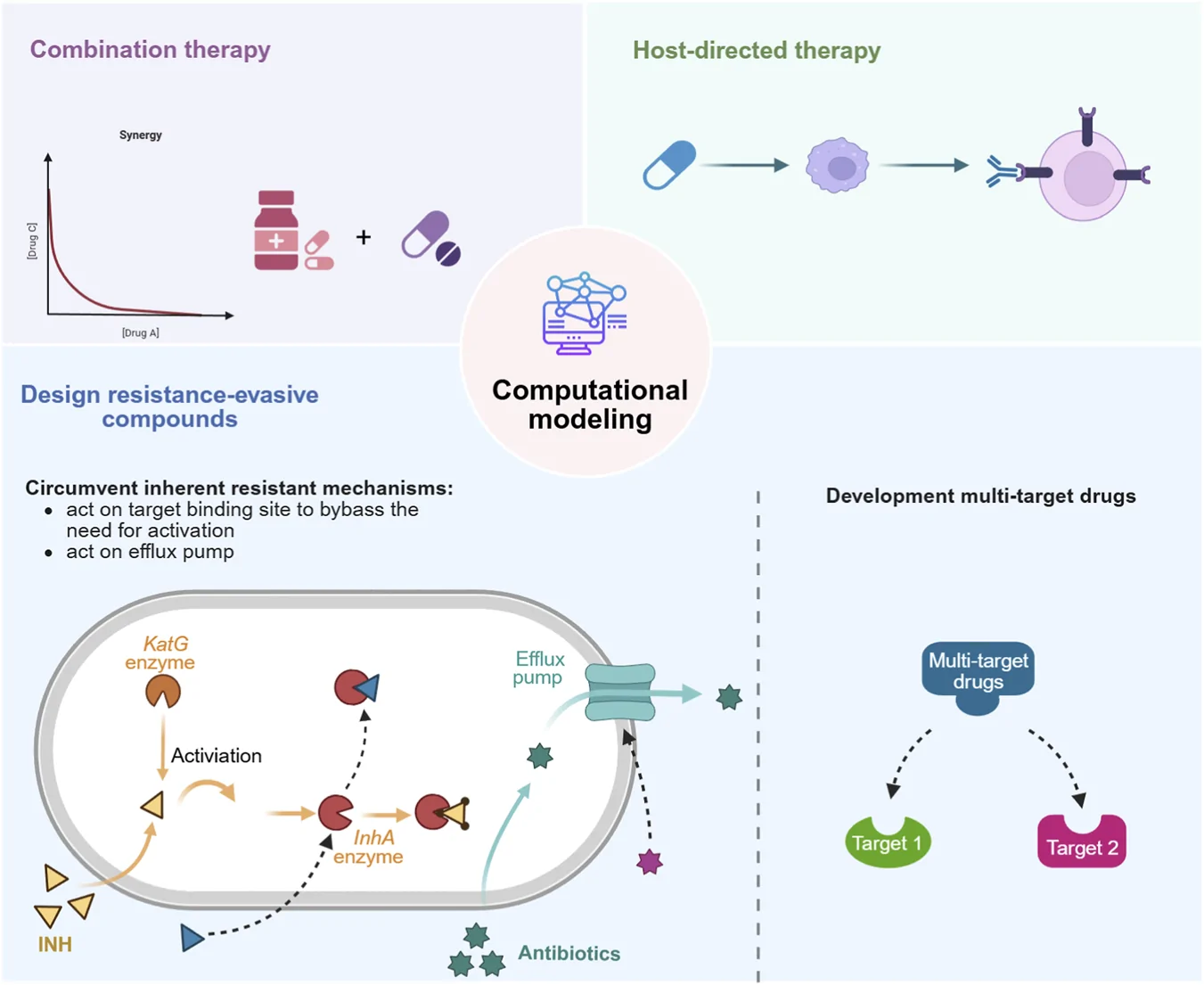
Computational modeling is applied to screen anti-TB compounds with activity against resistance through three key strategies: design of resistance-evasive compounds, host-directed therapy, and combination therapy.

### 4.1 Computational screening strategies against resistance

#### 4.1.1 Designing compounds to overcome resistance

The most common form of resistance in *Mtb* is acquired resistance, primarily arising from genetic mutations in drug targets, inactivation of prodrug-activating enzymes, or upregulation of efflux pumps (Farhat et al., 2024). Guided by these resistance mechanisms, computational modeling has been employed to specifically design and screen resistance-evasive compounds, aiming to mitigate the likelihood of resistance development.

One prominent strategy involves multi-target drug design, in which compounds simultaneously engage multiple proteins. Such approaches improve robustness against single-point mutations by maintaining activity through alternative, non-mutated targets, thereby reducing the likelihood of resistance development (Upadhayay et al., 2023). Multi-target design can be broadly categorized into two approaches. The first involves designing such compounds that interact with proteins across distinct biological pathways. These candidates are comprehensively evaluated through an integrative pipeline of molecular docking, bioactivity assays, and phenotypic screening (Battah et al., 2019; Venugopala et al., 2021). Alternatively, multi-target strategies may focus on multiple proteins within a single essential pathway. For instance, a hybrid computational workflow—comprising ligand-based modeling, molecular docking, and MD simulations—was applied to the Mur enzyme family involved in peptidoglycan biosynthesis. This approach enabled the identification of ChEMBL-derived compounds with favorable pharmacological profiles and potential multitarget activity (Kumari et al., 2023). Additionally, network pharmacology approaches have been employed to map interactions among key *Mtb* targets involved in growth and metabolism, guiding the prioritization of candidate inhibitors via docking studies (Singh et al., 2023).

Beyond multi-target strategies, another important direction is the development of direct-acting inhibitors that bypass the need for enzymatic activation. A notable example is INH, a prodrug requiring bioactivation by the catalase-peroxidase (*KatG*) enzyme to manifest its inhibitory effects on InhA. However, single nucleotide polymorphisms or insertions/deletions in *KatG* represent a well-documented cause of clinical INH resistance, rendering the drug ineffective (Lu et al., 2009; Zhao et al., 2006). To address this, direct-acting InhA inhibitors have been developed to circumvent *KatG*-mediated activation. For example, one study employed SBVS to assess a series of newly synthesized triclosan (TCN) derivatives targeting InhA (Armstrong et al., 2020). Given the unfavorable lipophilicity and poor oral bioavailability associated with the chlorine atom in TCN, analogs were rationally designed by replacing chlorine with alternative halogens. Virtual screening subsequently identified lead compounds with enhanced potency, solubility, and pharmacokinetic profiles (Chetty et al., 2021).

Efflux-mediated resistance presents another major obstacle to drug efficacy in *Mtb*, wherein overexpressed efflux pumps actively extrude therapeutic agents. Inhibiting these systems represents a viable strategy to restore intracellular drug concentrations and reinvigorate antimicrobial activity. For example, the *Rv1258c* gene encodes the Tap efflux pump, which is responsible for extruding antibiotics such as RIF. In the absence of experimentally resolved 3D structures for Tap, one study integrated homology modeling with molecular docking to successfully identify two natural product inhibitors with high predicted binding affinities, providing promising lead scaffolds to counteract efflux-mediated resistance (Biswas and Roy, 2024).

#### 4.1.2 Host-directed therapy

Host-directed therapy (HDT) seeks to bolster host immune defenses, compensate for immunological deficiencies, or inhibit bacterial survival mechanisms that rely on host-derived factors (de la Fuente-Nunez et al., 2023). By strengthening host factors rather than directly targeting the pathogen, HDT reduces selective pressure for resistance (Mehta et al., 2022). Computational modeling combined with experimental validation has facilitated the identification of immunomodulatory agents enhancing macrophage antimicrobial activity (Mishra et al., 2018; Nyambo et al., 2025). Pranlukast, identified from large chemical libraries, exemplifies such an agent with efficacy as monotherapy and adjunctive therapy alongside first-line TB drugs (Mishra et al., 2018). In another study, machine learning models trained on bioactivity data and *in vitro* screening results predicted numerous potential HDT candidates, with subsequent experimental confirmation of active compounds exhibiting host-directed anti-TB effects (Korbee et al., 2018).

#### 4.1.3 Combination therapy

Combination therapy remains a cornerstone of TB treatment by enhancing bactericidal efficacy and suppressing the emergence of drug resistance, and its design is increasingly supported by computational approaches that characterize pharmacodynamic and pharmacokinetic properties of drug combinations. At the pharmacodynamic level, MD simulations have revealed that drug–membrane interactions can modulate intracellular uptake, as exemplified by mefloquine-induced enhancement of antibacterial activity through increased membrane permeability (Dos Santos et al., 2021). Complementarily, SBVS have further identified compounds that modulate bacterial regulatory pathways, including quorum sensing inhibition, biofilm disruption, and host autophagy activation, thereby potentiating the activity of INH (Sarangi et al., 2024; Bi et al., 2025). More recently, one study integrating AlphaFold-predicted protein structures and molecular docking has expanded the search space of candidate synergistic combinations and enabled drug repurposing in complex clinical settings such as TB–diabetes comorbidity (Zhen et al., 2024).

However, such pharmacodynamic interaction alone is not sufficient to explain *in vivo* combination performance, as pharmacokinetic properties determine systemic and site-of-action drug exposure. Physiologically based pharmacokinetic modeling has therefore been used to quantify drug–drug interactions in TB regimens, including RIF-mediated reductions in bedaquiline exposure, while translational minimal physiologically based pharmacokinetic models have been used to predict lung and lesion-level drug concentrations and target attainment (Humphries et al., 2021; Mehta et al., 2023). Imaging-based studies of TB lesions have characterized spatial variability in drug distribution, including differential penetration into caseous necrotic regions, providing experimental evidence for heterogeneous site-of-infection exposure profiles (Prideaux et al., 2015).

### 4.2 The role of AI in resistance-oriented drug screening

AI has evolved from an auxiliary tool into an important component of resistance-oriented anti-TB drug discovery. AI techniques have contributed to notable advances across the entire drug discovery pipeline, from *de novo* drug design (Gangwal and Lavecchia, 2024), toxicity prediction (Tran et al., 2023) and drug resistance prediction (Cesaro et al., 2025) to the integration of end-to-end predictive platforms (Shen et al., 2024). In parallel, the advent of large language models (LLMs) tailored for biomedical research, such as DrugGPT (Li Y. et al., 2023) and BioGPT (Luo et al., 2022), has broadened the toolkit available for intelligent drug screening.

In resistance-related applications, AI has demonstrated distinct advantages in improving predictive accuracy, accelerating screening efficiency, and identifying structurally novel candidate compounds. One notable contribution lies in protein structure prediction, where AI has overcome several limitations of conventional homology modeling, which depends heavily on experimentally resolved homologous templates. AlphaFold, for instance, has significantly improved structural prediction accuracy. A comparative study involving caseinolytic protease B (ClpB), a key molecular chaperone associated with *Mtb* proteostasis and immune evasion, confirmed AlphaFold’s superior performance relative to conventional homology modeling approaches in structure-based applications (Singh et al., 2024). AI-driven screening has likewise demonstrated strong performance when applied to large-scale chemical libraries (Korbee et al., 2018). Notably, one AI-predicted compound exhibited a scaffold distinct from conventional antibiotics and displayed inhibitory activity against multiple bacterial pathogens beyond *Mtb* (Stokes et al., 2020). These findings exemplify the innovative potential of AI to uncover structurally novel bioactive molecules and to expand the chemical space available for TB drug screening against resistance.

Despite these advances, AI-driven modeling remains constrained by data quality, limited interpretability, and concerns regarding model reproducibility and generalizability. Consequently, neither AI-driven modeling nor hybrid computational approaches can fully substitute for experimental validation. Indeed, several studies have shown that hybrid pipelines integrating *in silico* prediction with *in vitro* validation yield more reliable results by reducing uncertainty and minimizing false-positive rates inherent to purely computational workflows (Veeravarapu et al., 2021; Thongdee et al., 2022).

## 5 Data resources for anti-TB drug screening

Data resources provide the foundation for computational anti-TB drug screening, influencing model reliability, chemical diversity, and predictive performance. These resources generally fall into two categories: general-purpose chemical databases and TB-specific repositories (Tables 4, 5).

**TABLE 4:** General-purpose databases for drug screening.

Resource name	Descriptions	Data counts	Websites	Types	Refs.
PubChem	Launched in 2004 as part of the Molecular Libraries Roadmap Initiative by the US National Institutes of Health (NIH), this public repository provides information on chemical substances and their biological activities.	119,319,203 compounds; 1,767,953 bioassays	https://pubchem.ncbi.nlm.nih.gov	Publicly accessible	Kim et al. (2021)
ChEMBL	Bioactive molecules exhibiting drug-like properties, this resource integrates chemical, bioactivity, and genomic data to support the translation of genomic insights into novel, effective drugs.	2,496,335 compounds; 16,003 targets; 15,500 drugs	https://www.ebi.ac.uk/chembl/	Publicly accessible	Zdrazil et al. (2024)
ChemDB	A chemical database housing nearly 5 million small molecules, each with predicted or experimentally determined physicochemical properties.	Nearly 5 million small molecules	https://cdb.ics.uci.edu/	Publicly accessible	Chen et al. (2007)
BindingDB	The first public molecular recognition database supports research, education and practice in drug discovery, pharmacology and related fields, containing experimental data on protein-small molecule interaction data.	About 3 million binding data for 9,354 proteins and over 1.3 million drug-like molecules.	https://www.bindingdb.org	Publicly accessible	Gilson et al. (2016)
CartBlanche22 (ZINC22)	A comprehensive collection of commercially available compounds, designed for virtual screening and drug discovery and provides access to a vast range of molecular structures for computational and experimental studies.	About 54.9 billion molecules in 2D and 5.9 billion in 3D structures	https://cartblanche22.docking.org/	Publicly accessible	Tingle et al. (2023)
DUD-E	An improved and revamped version of DUD (Directory of Useful Decoys), designed to benchmark molecular docking programs by offering challenging decoys.	22,886 active compounds and their affinities against 102 targets	http://dude.docking.org/	Publicly accessible	Mysinger et al. (2012)
DrugBank	A comprehensive online database that provides information on drugs and drug targets.	17,445 drug entries: 2,813 approved small molecule drugs, 1,678 approved biologics, 135 nutraceuticals, and over 6,731 experimental drugs	https://go.drugbank.com/	Partially free-to-access	Knox et al. (2024)
Enamine REAL	A classical database of enumerated structures, serving as a tool for discovering new hit molecules through large-scale virtual screening and finding analogs of these hit molecules.	About 5.5 billion compounds and more than 11 billion drug-like compounds	https://enamine.net/compound-collections/real-compounds/real-database	Commercially available	Shivanyuk et al. (2007)
NPASS	Its content is searchable by keywords, physicochemical properties, structural similarity, species, and target.	94,481 unique natural products with biologically evaluated and together with 958,866 activity records on 7,753 targets	http://bidd.group/NPASS	Publicly accessible	Zhao et al. (2023)

**TABLE 5:** Specialized databases for drug screening to combat tuberculosis.

Resource name	Descriptions	Websites
TBDB	An integrated database offering access to TB genomic data and resources, essential for the discovery and development of TB drugs, vaccines, and biomarkers.	http://www.tbdb.org/
TuberQ	A bioinformatics database designed to predict and analyze protein-ligand interactions in plant systems, focusing on tuber proteins. It provides tools for in-depth exploration of protein-ligand binding and functional annotation, along with examples and resources for practical application.	http://tuberq.proteinq.com.ar/bioflux/information/UsageExample.html
Mycobrowser	A comprehensive genomic and proteomic repository for pathogenic mycobacteria, offering manually curated annotations and tools to support the genomic and proteomic study of these organisms.	https://mycobrowser.epfl.ch/
TB Drug Target Database	It contains information on antitubercular drugs and their target proteins for TB treatment. Details on the drugs, including their structural information, are provided, along with analysis results of the target proteins.	https://www.bioinformatics.org/tbdtdb/
TIBLE	A web-based, freely accessible resource offers comprehensive data on minimal inhibitory concentrations of small molecules against various mycobacterial species, along with predicted target and off-target interactions specific to *Mycobacterium tuberculosis*.	http://www-cryst.bioc.cam.ac.uk/tible/
NIAID TB Portals	A repository containing socioeconomic/geographic, clinical, laboratory, radiological, and genomic data from patient cases of drug-resistant tuberculosis.	https://tbportals.niaid.nih.gov/

General-purpose databases, including PubChem (Kim et al., 2021), ChEMBL (Zdrazil et al., 2024), ChemDB (Chen et al., 2007), BindingDB (Gilson et al., 2016), ZINC (Tingle et al., 2023), and DUD-E (Mysinger et al., 2012), are freely accessible and extensively utilized in virtual screening workflows. DrugBank, a partially open-access platform, integrates comprehensive data on drug-target interactions, pharmacokinetics, and approved drug annotations, making it particularly valuable for drug repurposing and target prioritization (Knox et al., 2024). Commercial libraries such as Enamine REAL further expand accessible chemical space and are particularly well-suited for deep-learning-based generative models (Shivanyuk et al., 2007). Natural-product databases such as NPASS additionally provide chemically diverse scaffolds relevant to anti-TB discovery (Zhao et al., 2023). Complementing these resources, TB-specific databases curate information directly related to *Mtb*, including resistance-associated mutations, essential genes, and validated therapeutic targets. Such resources facilitate the rational design of agents that can counteract drug resistance.

Despite substantial progress, data accessibility remains a significant challenge. Many large-scale compound libraries remain proprietary, limiting reproducibility and open innovation. Collaborative initiatives such as TB Portals partially address this limitation by providing openly accessible clinical, genomic, and radiological datasets that support global computational TB research (Rosenthal et al., 2017).

## 6 Discussion

Despite substantial advances in VS, AI-driven modeling, and hybrid computational approaches, a persistent translational gap remains between *in silico* predictions and clinically effective anti-TB therapies. This gap is not attributable to isolated methodological shortcomings but rather reflects a systematic mismatch between target-centric computational workflows and the multi-scale biological and pharmacological complexity of *Mtb*. To provide an integrative overview of how current computational strategies relate to TB-specific constraints and translational requirements, these relationships are summarized in Figure 5.

**FIGURE 5:**
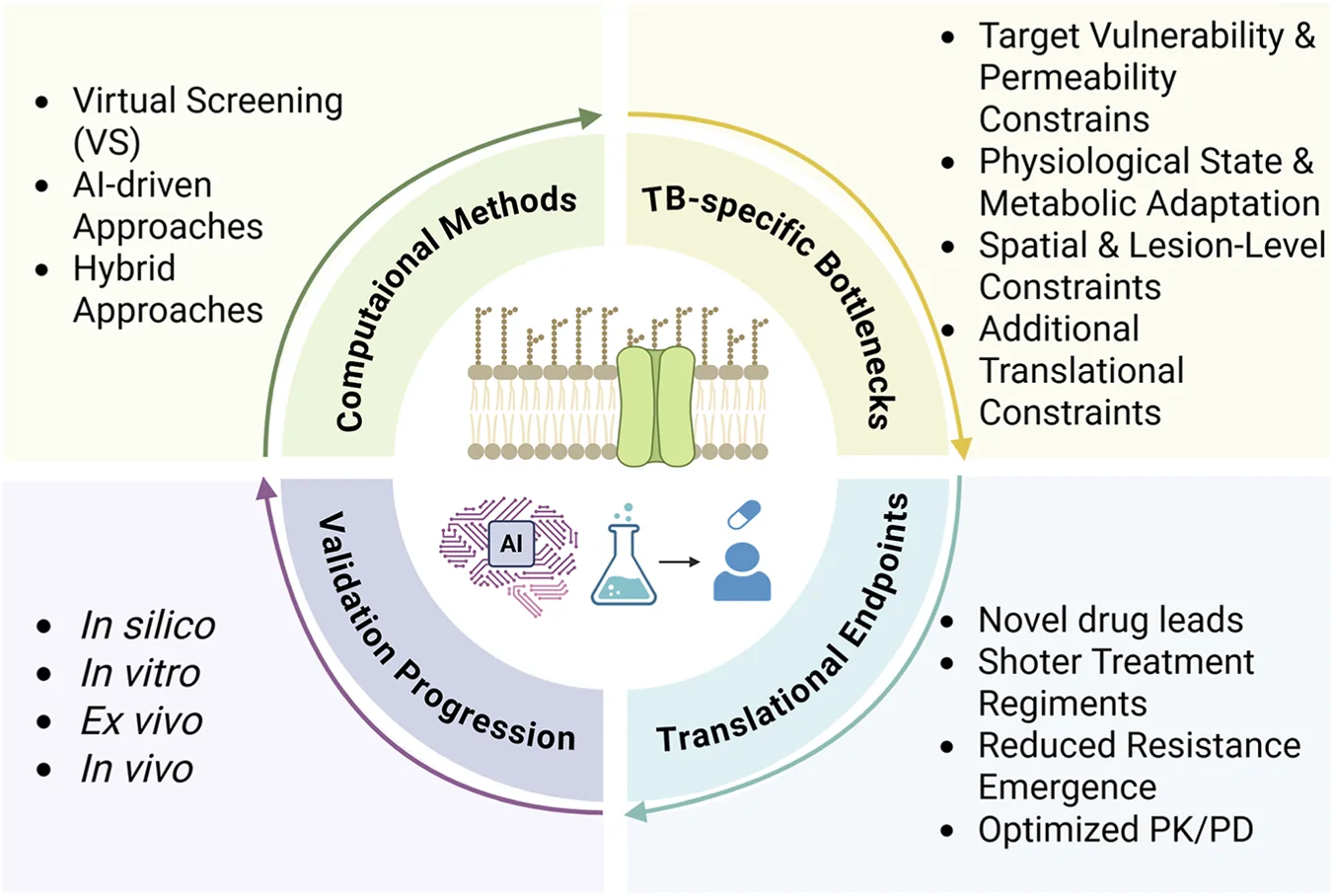
Integrative framework linking computational methods, TB-specific bottlenecks, validation progression, and translational endpoints in drug discovery. Figure created with www.BioRender.com.

At the molecular level, target essentiality does not necessarily translate into pharmacological vulnerability due to resistance mutations and structural constraints. Moving to the cellular level, the mycobacterial cell envelope and efflux systems routinely decouple binding affinity from intracellular target engagement (Radchenko et al., 2023). This misalignment deepens at the physiological level, where metabolic adaptation and non-replicating states further compromise the translational predictive value of biochemical potency (Mashabela et al., 2019). This vulnerability exposes a systemic replication bias in current CADD workflows, which remain largely optimized for actively replicating bacilli and may therefore inadequately capture metabolically quiescent latent subpopulations. Therapeutic interest in latent and non-replicating *Mtb* has consequently shifted attention toward physiological adaptation and persistence-associated vulnerabilities that are unlikely to be identified through conventional growth-dependent screening paradigms alone (Baral et al., 2024; Eke et al., 2023). In addition, lesion-level spatial heterogeneity and limited drug penetration create pharmacokinetic–pharmacodynamic mismatches that are rarely represented in current CADD pipelines (Prideaux et al., 2015). Collectively, these multi-scale bottlenecks remain severely underrepresented in existing computational discovery pipelines, which continue to operate under a restrictive, target-centric paradigm rather than embracing cellular, physiological, and lesion-level constraints.

Beyond these biological barriers, a more fundamental limitation lies in the mismatch between single-target optimization and the regimen-based TB therapy. Current clinical treatment relies on multi-drug combinations with coordinated pharmacological interactions to suppress resistance and achieve sterilizing activity, particularly in drug-resistant TB (Larkins-Ford et al., 2022). However, most computational approaches remain focused on identifying individual high-affinity inhibitors, without explicitly considering combination-level performance or treatment context. As a result, current *in silico* frameworks capture only a fraction of the determinants governing clinical outcomes.

Collectively, these considerations suggest that current anti-TB drug discovery remains constrained by a fundamental mismatch between target-centric computational optimization and the multi-scale, regimen-driven nature of TB therapy. Addressing this gap requires a shift toward a more integrative framework that accounts for lesion penetration, intracellular and non-replicating bacilli activity, and drug–drug interaction effects, which are currently underrepresented in existing computational pipelines. This limitation highlights a critical barrier to translating *in silico* predictions into clinically relevant outcomes.

## 7 Conclusion and future perspectives

TB drug discovery remains hindered by a persistent translational bottleneck: despite significant advances in computational modeling, these innovations have yet to yield clinically approved therapies for drug-resistant *Mtb*. While VS, AI-driven modeling, and hybrid computational frameworks have drastically expanded the accessible chemical space and enhanced early-stage hit identification, their ultimate clinical impact remains limited.

Overcoming this limitation requires a fundamental shift in the computational drug discovery paradigm, beginning with the fortification of our underlying data infrastructure. Developing robust, standardized, and comprehensively annotated datasets is essential to enhance model generalizability and mitigate bias. By harmonizing assay formats, activity thresholds, and resistance profiles, the field can enable reliable cross-study comparisons and facilitate the training of large-scale models. Building upon this refined data, mechanistic biological insights must be more explicitly integrated into computational workflows. Future models cannot rely solely on static target-binding predictions; rather, they must incorporate the vital determinants of *in vivo* efficacy. Embedding multi-scale constraints—such as cell envelope permeability, efflux pump dynamics, metabolic heterogeneity, and lesion-level pharmacokinetics—will be crucial for elevating the translational relevance of early-stage discoveries. In parallel, AI-driven models must evolve beyond isolated predictive tasks. By embedding AI approaches within biologically informed frameworks, we can achieve true integration across the molecular, cellular, and pharmacological scales that dictate TB pathology. Ultimately, this biological context must be scaled up to reflect clinical reality, necessitating a pivot toward regimen-aware, systems-level modeling. Because TB treatment is intrinsically dependent on combination therapy, future computational strategies should prioritize drug-drug interactions, synergistic efficacy, and resistance suppression over the exclusive optimization of single compounds.

Translating computational drug screening into clinically effective therapies for drug-resistant TB depends on closely aligning *in silico* methodologies with the hierarchical biology of *Mtb* infection. Future computational drug screening frameworks should move beyond molecule-centric predictions toward biologically informed, resistance-aware, and regimen-oriented strategies that explicitly account for resistance mechanisms, combination therapy, and clinical treatment complexity. Such advances will be essential for accelerating the identification of effective therapeutics against MDR- and XDR-TB.
